# Supplementary material for: Nutrient and salt depletion synergistically boosts glucose metabolism in individual Escherichia coli cells
Source: Commun Biol. 2022 Apr 20;5:385. doi: 10.1038/s42003-022-03336-6 (PMC9021252; doi:10.1038/s42003-022-03336-6)
Supplement: Supplementary file 1 — Supplemental material [file 42003_2022_3336_MOESM1_ESM.pdf]

SUPPLEMENTARY INFORMATION

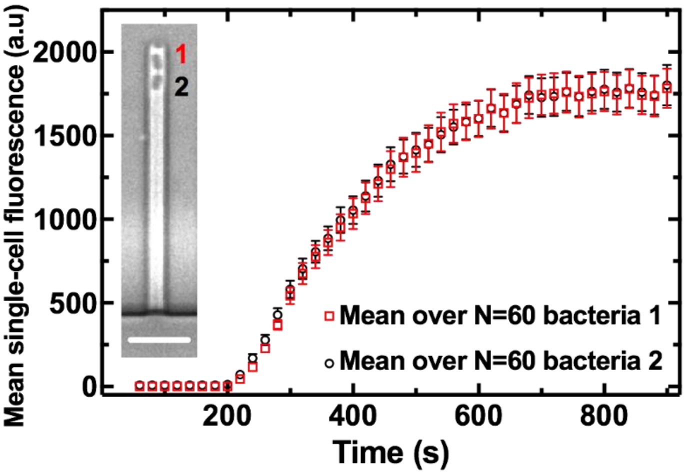

Supplementary Figure 1. Temporal dependence of the mean intracellular fluorescence of the glucose analogue 2-NBDG averaged over 60 individual *E. coli* at the top of each dead-end bacteria-hosting channel (red squares, position 1) or below such cells (black circles, position 2). Bacteria at position 2 are closer to the main microfluidic chamber i.e. the 2-NBDG source, whereas bacteria at position 1 are screened by one cell. Statistical comparisons are reported in Table S1. Inset: bright-field microscopy image illustrating bacteria in positions 1 and 2, scale bar: 5 µm.

Time (s)	t ratio	df	*p*-value
0			
100	1,26	58	0,21
200	1,60	58	0,11
300	0,52	58	0,60
400	0,18	58	0,86
500	0,12	58	0,91
600	0,01	58	0,99
700	0,11	58	0,92
800	0,07	58	0,94
900	0,11	58	0,91

Supplementary Table 1. Statistical comparisons of 2-NBDG accumulation in *E. coli* at the top of each dead-end bacteria-hosting channel or below such cells (positions 1 and 2 in Fig. S1, respectively).

Time (s)	Optimal growth conditions		Nutrient depletion	
	Mean fluorescence (a.u.)	CV fluorescence (%)	Mean fluorescence (a.u.)	CV fluorescence (%)
0	0	0	0	0
100	17	48	22	61
200	37	90	40	49
300	250	46	417	58
400	435	35	546	58
500	533	33	647	55
600	613	32	692	57
700	685	32	762	55
800	699	33	769	58
900	733	32	740	60
1000	730	34	683	54
1100	761	32	663	59
1200	580	46	458	54
1300	350	47	293	61
1400	231	49	208	64
1500	156	52	150	67
1600	112	52	113	69
1700	83	55	91	67
1800	63	55	76	69
1900	53	53	64	64
2000	42	47	51	64
Time (s)	Salt depletion		Nutrient and salt depletion	
	Mean fluorescence (a.u.)	CV fluorescence (%)	Mean fluorescence (a.u.)	CV fluorescence (%)
0	0	0	0	0
100	12	86	12	86
200	13	84	16	90
300	148	70	722	43
400	262	45	1208	42
500	298	43	1507	40
600	332	39	1669	38
700	326	41	1648	39
800	317	43	1676	38
900	373	39	1714	38
1000	393	43	1583	39
1100	429	38	1670	38
1200	328	45	1146	46
1300	155	38	582	55
1400	83	50	341	64
1500	44	98	186	55
1600	34	118	124	60
1700	23	128	82	79
1800	25	143	53	70
1900	29	151	40	62
2000	18	169	27	71

Supplementary Table 2. Temporal dependence of the mean and coefficient of variation of intracellular fluorescence of the glucose analogue 2-NBDG over at least 30 individual *E. coli* (collated from biological triplicate) per environmental condition as detailed in Figure 2.

Time (s)	Nutrient depletion		
	**t ratio**	**df**	***p*-value**
0			
100	2,07	112,00	0,04
200	0,45	112,00	0,65
300	4,96	112,00	$<10^{-5}$
400	2,53	112,00	0,01
500	2,27	112,00	0,03
600	1,43	112,00	0,15
700	0,30	112,00	0,20
800	1,11	112,00	0,27
900	0,12	112,00	0,90
**Time (s)**	**Salt depletion**		
	**t ratio**	**df**	***p*-value**
0			
100	3,46	166,00	$<10^{-3}$
200	6,43	166,00	$<10^{-6}$
300	5,89	166,00	$<10^{-6}$
400	7,94	166,00	$<10^{-6}$
500	9,47	166,00	$<10^{-6}$
600	10,74	166,00	$<10^{-6}$
700	12,05	146,00	$<10^{-6}$
800	12,35	146,00	$<10^{-6}$
900	11,11	146,00	$<10^{-6}$
**Time (s)**	**Nutrient and salt depletion**		
	**t ratio**	**df**	***p*-value**
0			
100	3,39	120,00	$<10^{-3}$
200	3,99	120,00	$<10^{-4}$
300	11,91	120,00	$<10^{-6}$
400	12,33	120,00	$<10^{-6}$
500	13,14	120,00	$<10^{-6}$
600	13,55	120,00	$<10^{-6}$
700	11,89	110,00	$<10^{-6}$
800	11,96	110,00	$<10^{-6}$
900	11,60	110,00	$<10^{-6}$

Supplementary Table 3. Statistical comparisons of 2-NBDG accumulation in *E. coli* under nutrient, salt or combined nutrient and salt depletion compared to optimal growth conditions.

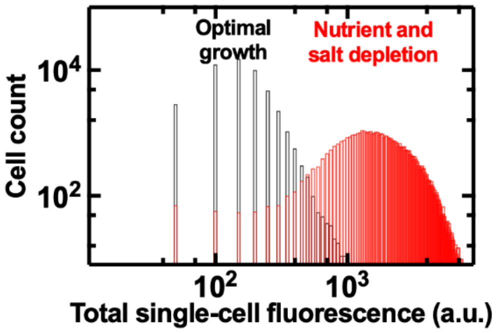

Supplementary Figure 2. Total intracellular fluorescence of the glucose analogue 2-NBDG in individual *E. coli* under optimal growth conditions and combined nutrient and salt depletion (black and red bars, respectively) measured by flow cytometry after 900s bulk incubation in 2-NBDG. Noteworthy, these measurements were not normalized by cell size.

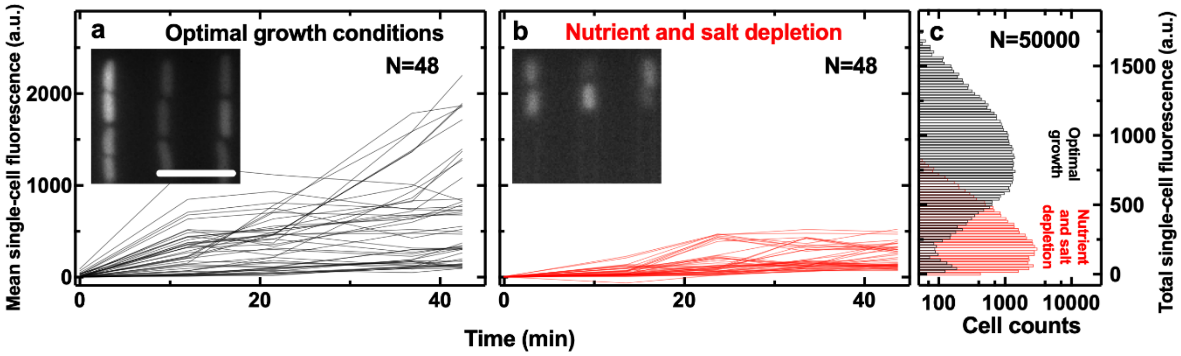

Supplementary Figure 3. Temporal dependence of the mean intracellular fluorescence of thioflavin T in individual *E. coli* in **a)** optimal growth conditions and **b)** under combined nutrient and salt depletion. Lines are temporal dependences of the intracellular fluorescence of individual bacteria collated from biological triplicate. Noteworthy, we measured thioflavin T fluorescence as the mean fluorescent values of each pixel constituting each bacterium, thus normalizing by cell size. Insets: corresponding fluorescence images at t=45min when the intracellular ThT accumulation has reached saturation levels in individual bacteria. **c)** Corresponding total intracellular fluorescence of thioflavin T under optimal growth conditions or combined nutrient and salt depletion (black and red bars, respectively) measured by flow cytometry after 45min bulk incubation in thioflavin T. Noteworthy, these measurements were not normalized by cell size. Statistical comparisons are reported in Table S4.

Time (min)	Microfluidics-microscopy assay			Flow cytometry assay		
	t ratio	df	*p*-value	t ratio	df	*p*-value
0	3,78	94	$<10^{-6}$			
12	7,98	94	$<10^{-6}$			
22	5,56	94	$<10^{-6}$			
37	6,01	94	$<10^{-6}$			
42	6,23	94	$<10^{-6}$	257,9	99578	$<10^{-6}$

Supplementary Table 4. Statistical comparisons of thioflavin T accumulation in *E. coli* in optimal growth conditions and under combined nutrient and salt depletion as measured via single-cell microfluidics-microscopy and flow cytometry.

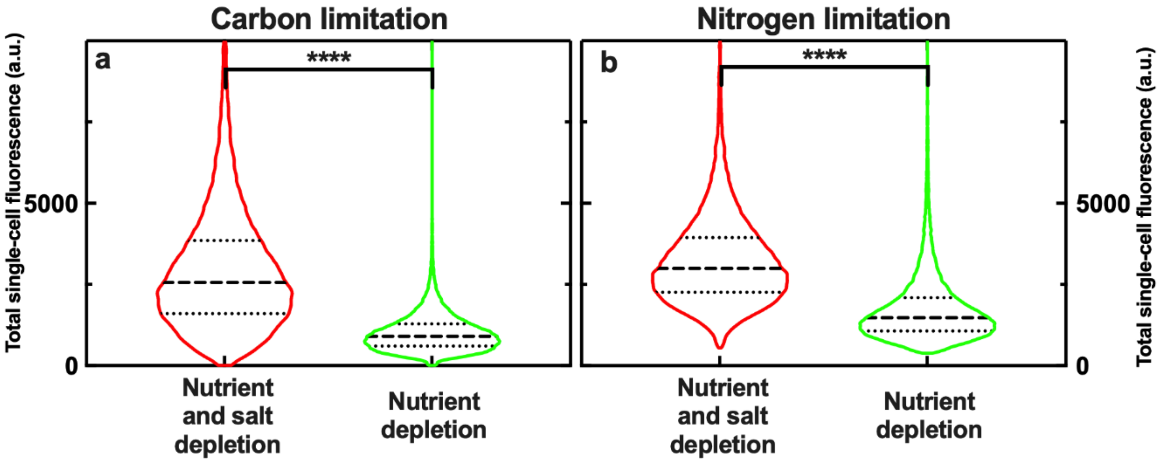

Supplementary Figure 4. Distribution of total intracellular fluorescence of 2-NBDG under nutrient depletion alone or combined nutrient and salt depletion (green and red violins, respectively) using M9 minimal medium with limited (i.e. 0.1 g/L) **a)** glucose or **b)** ammonia. Measurements were performed on 50,000 bacteria for each environmental condition using flow cytometry after 900s bulk incubation in 2-NBDG. These measurements were not normalized by cell size. ****: p-value <0.0001.

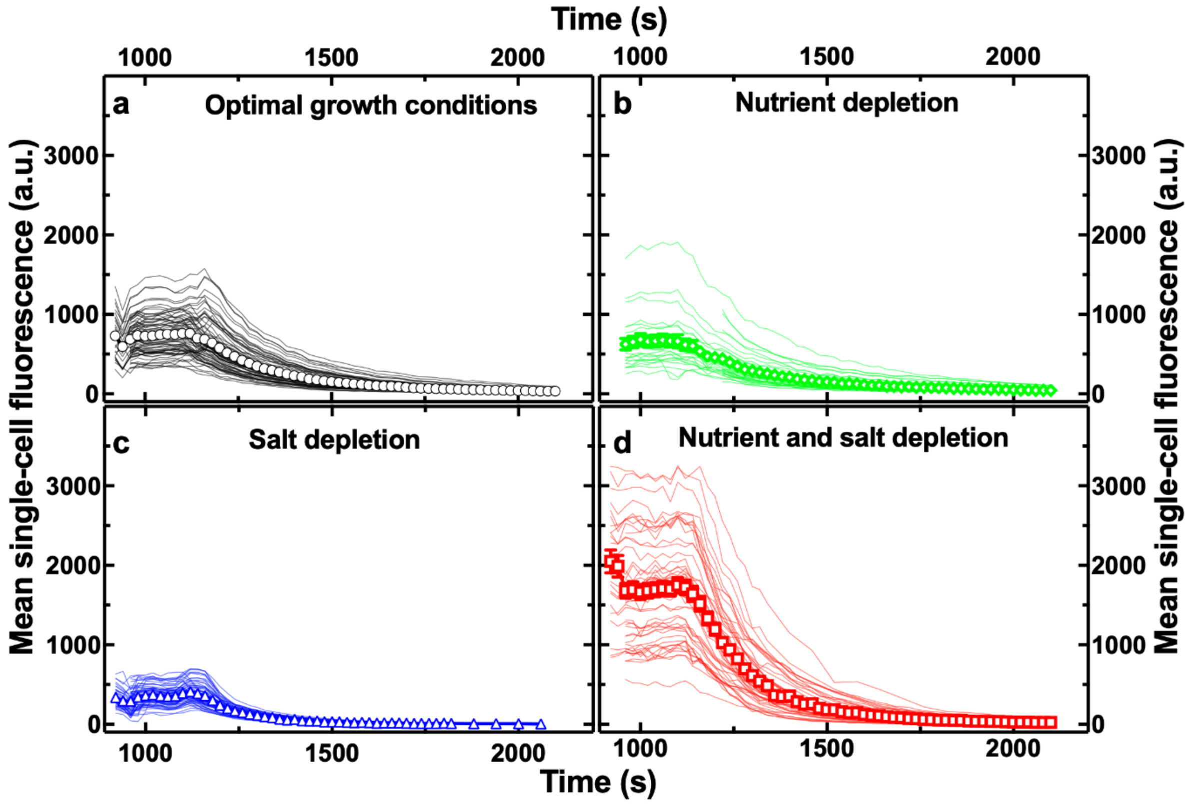

Supplementary Figure 5. Temporal dependence of the mean intracellular fluorescence of the glucose analogue 2-NBDG in individual *E. coli* under **a)** optimal growth conditions, **b)** nutrient depletion, **c)** salt depletion or **d)** combined nutrient and salt depletion during removal of 2-NBDG from the extracellular environment. Lines are temporal dependences of the intracellular fluorescence of individual bacteria from biological triplicate. Symbols and error bars are the corresponding means and standard error of the means of such single-cell measurements. Means and coefficient of variations of these single-cell values are reported in Table S2. These measurements were normalized by cell size. Measurements were carried out on N=76, 38, 90 and 46 individual bacteria, in a)-d), respectively.

Comparisons with optimal growth	Uptake rate			Degradation rate		
	t ratio	df	*p*-value	t ratio	df	*p*-value
Nutrient depletion	3.88	62,0	0.0003	3.41	42	0.0015
Salt depletion	7.46	116,0	<0.0001	10.97	83	<0.0001
Nutrient and salt depletion	2.59	86,0	0.01	9.30	80	<0.0001

Supplementary Table 5. Statistical comparisons of the predicted 2-NBDG uptake and degradation values under nutritional, salinity or combined nutritional and salinity depletion compared to optimal growth conditions.

Environment	Uptake rate		Degradation rate	
	Median	CV (%)	Median	CV (%)
Optimal growth conditions	-1,73	13	-2,35	3
Nutrient depletion	-1,94	15	-2,31	9
Salt depletion	-2,05	15	-2,12	9
Nutrient and salt depletion	-1,52	19	-2,18	5

Supplementary Table 6. Median and coefficient of variation (CV) of the predicted uptake and degradation rate values in optimal growth conditions, under nutritional, salinity or combined nutritional and salinity depletion.

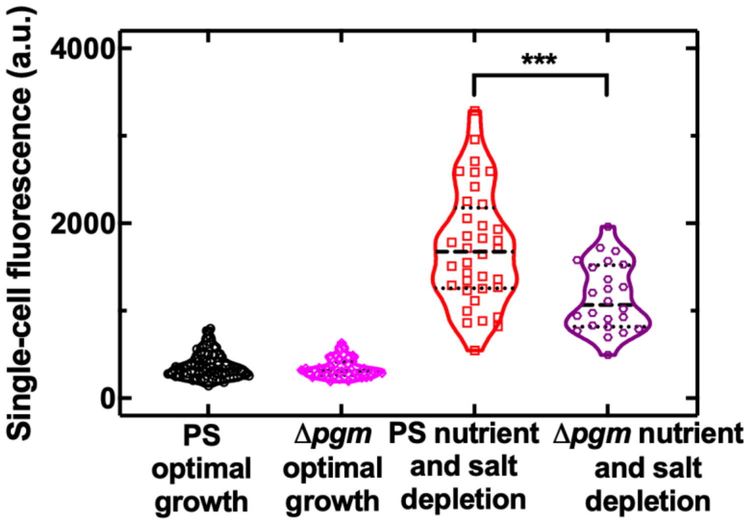

Supplementary Figure 6. Distribution of single-cell fluorescence after 900 s incubation in 2-NBDG for the parental strain (PS) and Δ*pgm* deletion mutant under salt depletion or simultaneous nutrient and salt depletion. Dashed and dotted lines indicate the median and quartiles of each distribution, respectively. Under nutrient and salt depletion the Δ*pgm* deletion mutant displayed significantly lower 2-NBDG accumulation compared to the parental strain (***). In contrast, under optimal growth conditions the Δ*pgm* deletion mutant displayed 2-NBDG accumulation comparable to the parental strain.

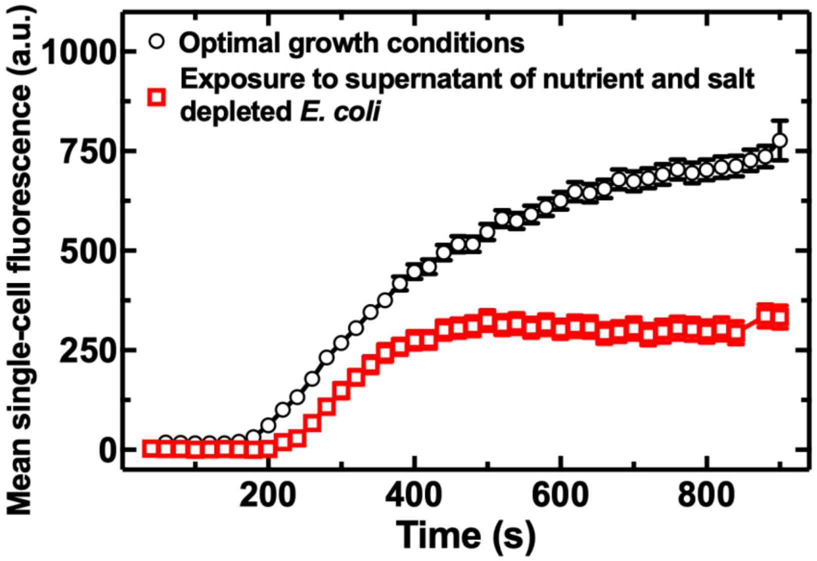

Supplementary Figure 7. Temporal dependence of the mean intracellular fluorescence of the glucose analogue 2-NBDG in *E. coli* cultured in optimal growth conditions without (circles) or with an additional 1h exposure to the supernatant collected from *E. coli* cultures under combined nutritional and salinity depletion (squares) before 2-NBDG accumulation measurements. Symbols and error bars are the means and standard error of the means over at least 20 single-cell measurements.

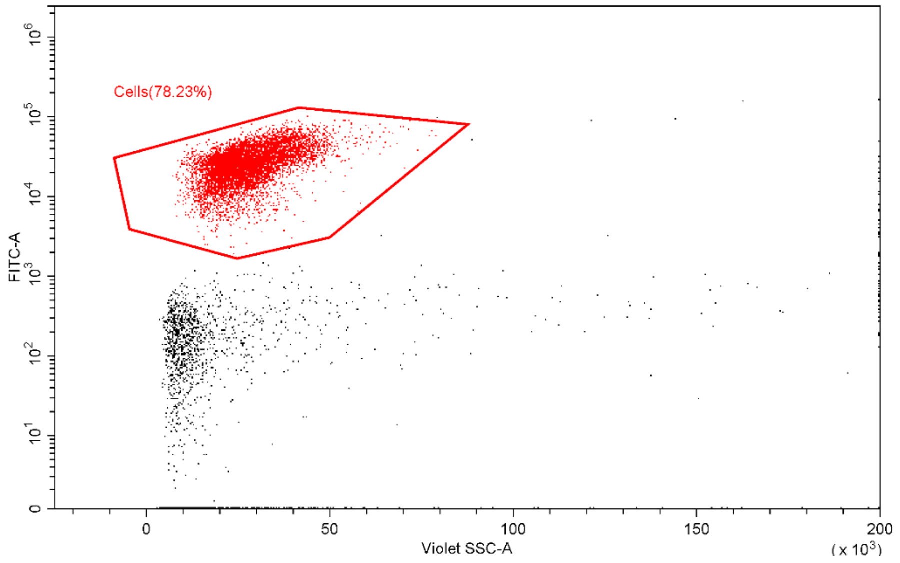

Supplementary Figure 8. Gating strategy for all flow cytometry experiments. Cells (red) were separated from background debris (black) by gating bacterial cells (red) using a plot of fluorescein isothiocyanate channel (FITC-A) against violet side scatter (Violet-SSC-A).
