# Supplementary material for: Nutrient and salt depletion synergistically boosts glucose metabolism in individual Escherichia coli cells
Source: Commun Biol. 2022 Apr 20;5:385. doi: 10.1038/s42003-022-03336-6 (PMC9021252; doi:10.1038/s42003-022-03336-6)
Supplement: Supplementary file 2 — Description of Additional Supplementary Files [file 42003_2022_3336_MOESM2_ESM.pdf]

Description of Additional Supplementary Files

File name: Supplementary Data 1
Description: RNA transcript log_2 fold change and adjusted p-values file 1

File name: Supplementary Data 2
Description: RNA transcript log_2 fold change and adjusted p-values file 2

File name: Supplementary Data 3
Description: Protein log_2 fold change and adjusted p-values

File name: Supplementary Data 4
Description: Phosphoproteomic modifications and abundance

File name: Supplementary Data 5
Description: Exact p-values for main manuscript figures

File name: Supplementary Data 6
Description: All raw data for main manuscript figures
