# Supplementary material for: Nutrient and salt depletion synergistically boosts glucose metabolism in individual Escherichia coli cells
Source: Commun Biol. 2022 Apr 20;5:385. doi: 10.1038/s42003-022-03336-6 (PMC9021252; doi:10.1038/s42003-022-03336-6)
Supplement: Supplementary file 9 — Reporting Summary [file 42003_2022_3336_MOESM9_ESM.pdf]

nature portfolio

Corresponding author(s): Stefano Pagliara

Last updated by author(s): Mar 3, 2022

Reporting Summary

Nature Portfolio wishes to improve the reproducibility of the work that we publish. This form provides structure for consistency and transparency in reporting. For further information on Nature Portfolio policies, see our Editorial Policies and the Editorial Policy Checklist.

Statistics

For all statistical analyses, confirm that the following items are present in the figure legend, table legend, main text, or Methods section.

n/a	Confirmed	
☐	☒	The exact sample size (n) for each experimental group/condition, given as a discrete number and unit of measurement
☐	☒	A statement on whether measurements were taken from distinct samples or whether the same sample was measured repeatedly
☐	☒	The statistical test(s) used AND whether they are one- or two-sided<br>*Only common tests should be described solely by name; describe more complex techniques in the Methods section.*
☒	☐	A description of all covariates tested
☐	☒	A description of any assumptions or corrections, such as tests of normality and adjustment for multiple comparisons
☐	☒	A full description of the statistical parameters including central tendency (e.g. means) or other basic estimates (e.g. regression coefficient) AND variation (e.g. standard deviation) or associated estimates of uncertainty (e.g. confidence intervals)
☐	☒	For null hypothesis testing, the test statistic (e.g. F, t, r) with confidence intervals, effect sizes, degrees of freedom and P value noted<br>*Give P values as exact values whenever suitable.*
☐	☒	For Bayesian analysis, information on the choice of priors and Markov chain Monte Carlo settings
☒	☐	For hierarchical and complex designs, identification of the appropriate level for tests and full reporting of outcomes
☐	☒	Estimates of effect sizes (e.g. Cohen's d, Pearson's r), indicating how they were calculated

Our web collection on statistics for biologists contains articles on many of the points above.

Software and code

Policy information about availability of computer code

Data collection	LabView<br>CytExpert 2.4<br>CLARIOstar Plus V6.00 R2<br>Xcalibur 3.0
Data analysis	ImageJ 1.8.0_172<br>GraphPad Prism V9<br>R 4.1.2<br>Proteome Discoverer v2.1<br>Matlab R2018

For manuscripts utilizing custom algorithms or software that are central to the research but not yet described in published literature, software must be made available to editors and reviewers. We strongly encourage code deposition in a community repository (e.g. GitHub). See the Nature Portfolio guidelines for submitting code & software for further information.

Data

Policy information about availability of data

All manuscripts must include a data availability statement. This statement should provide the following information, where applicable:

- Accession codes, unique identifiers, or web links for publicly available datasets
- A description of any restrictions on data availability
- For clinical datasets or third party data, please ensure that the statement adheres to our policy

All RNA sequencing data and proteomics data is available in Supplementary Data 1-4. Exact p-values, where shown on Fig. 1-5, are available in Supplementary Data 5. The source data underlying Figs. 1-5 are provided as Supplementary Data 6. Any other relevant data are available upon reasonable request.

Field-specific reporting

Please select the one below that is the best fit for your research. If you are not sure, read the appropriate sections before making your selection.

☒ Life sciences ☐ Behavioural & social sciences ☐ Ecological, evolutionary & environmental sciences

For a reference copy of the document with all sections, see nature.com/documents/nr-reporting-summary-flat.pdf

Life sciences study design

All studies must disclose on these points even when the disclosure is negative.

Sample size	Sample sizes were determined based on similar experiments and prior experience from our collaborating laboratories. No statistical method was used to predetermine sample sizes
Data exclusions	No data was excluded.
Replication	Biological triplicates were universally used. The number of replicates for each specific experiment is also indicated in the manuscript and figure legends.
Randomization	Randomization is not relevant to the current study.
Blinding	Blinding is not relevant to the current study.

Reporting for specific materials, systems and methods

We require information from authors about some types of materials, experimental systems and methods used in many studies. Here, indicate whether each material, system or method listed is relevant to your study. If you are not sure if a list item applies to your research, read the appropriate section before selecting a response.

Materials & experimental systems

n/a	Involved in the study	
☒	☐	Antibodies
☒	☐	Eukaryotic cell lines
☒	☐	Palaeontology and archaeology
☒	☐	Animals and other organisms
☒	☐	Human research participants
☒	☐	Clinical data
☒	☐	Dual use research of concern

Methods

n/a	Involved in the study	
☒	☐	ChIP-seq
☐	☒	Flow cytometry
☒	☐	MRI-based neuroimaging

Flow Cytometry

Plots

Confirm that:

☒ The axis labels state the marker and fluorochrome used (e.g. CD4-FITC).

☒ The axis scales are clearly visible. Include numbers along axes only for bottom left plot of group (a 'group' is an analysis of identical markers).

☒ All plots are contour plots with outliers or pseudocolor plots.

☒ A numerical value for number of cells or percentage (with statistics) is provided.

Methodology

Sample preparation	1 mL of E. coli liquid overnight culture was aliquoted into an Eppendorf at t=3 or 17 h and spun down at 13,400 rpm for 5 min using a microcentrifuge (SLS Lab Basics, UK). For uptake experiments, the bacterial pellet was resuspended in 1 mL of fluorescent substrate solution; either 30 µM 2-NBDG or 50 µM ThT and incubated for 15 or 45 min, respectively. After incubation, cells were pelleted at 13,400 rpm for 5 min using a table top centrifuge and resuspended in 1 ml of phosphate buffer solution (PBS) and further diluted as necessary.
Instrument	Beckman Coulter CytoFLEX S
Software	Data were initially collected using CytExpert software, exported and later processed using GraphPad Prism 9.
Cell population abundance	n/a
Gating strategy	Cells were separated from background debris by gating bacterial cells using a plot of fluorescein isothiocyanate channel (FITC-A) against violet side scatter (Violet-SSC-A).

☒ Tick this box to confirm that a figure exemplifying the gating strategy is provided in the Supplementary Information.
